# Genetic metabolic complementation establishes a requirement for GDP-fucose in *Leishmania*

**DOI:** 10.1074/jbc.M117.778480

**Published:** 2017-05-02

**Authors:** Hongjie Guo, Natalia M. Novozhilova, Giulia Bandini, Salvatore J. Turco, Michael A. J. Ferguson, Stephen M. Beverley

**Affiliations:** From the ‡ Department of Molecular Microbiology, Washington University School of Medicine, St. Louis, Missouri 63110,; the § Department of Biochemistry, University of Kentucky Medical Center, Lexington, Kentucky 40536, and; the ¶ Division of Biological Chemistry and Drug Discovery, School of Life Science, University of Dundee, Dundee DD1 5EH, Scotland, United Kingdom

**Keywords:** adhesin, glycoconjugate, Leishmania, parasite metabolism, protozoan, virulence factor, D-Arabinopyranose, Fucose, Trypanosomatidae, nucleotide sugar

## Abstract

To survive in its sand fly vector, the trypanosomatid protozoan parasite *Leishmania* first attaches to the midgut to avoid excretion, but eventually it must detach for transmission by the next bite. In *Leishmania major* strain Friedlin, this is controlled by modifications of the stage-specific adhesin lipophosphoglycan (LPG). During differentiation to infective metacyclics, d-arabinopyranose (d-Ara*p*) caps the LPG side-chain galactose residues, blocking interaction with the midgut lectin PpGalec, thereby leading to parasite detachment and transmission. Previously, we characterized two closely related *L. major* genes (*FKP40* and *AFKP80*) encoding bifunctional proteins with kinase/pyrophosphorylase activities required for salvage and conversion of l-fucose and/or d-Ara*p* into the nucleotide-sugar substrates required by glycosyltransferases. Whereas only AFKP80 yielded GDP-d-Ara*p* from exogenous d-Ara*p*, both proteins were able to salvage l-fucose to GDP-fucose. We now show that Δ*afkp80*^−^ null mutants ablated d-Ara*p* modifications of LPG as predicted, whereas Δ*fkp40*^−^ null mutants resembled wild type (WT). Fucoconjugates had not been reported previously in *L. major*, but unexpectedly, we were unable to generate *fkp40*^−^/*afkp80*^−^ double mutants, unless one of the A/FKPs was expressed ectopically. To test whether GDP-fucose itself was essential for *Leishmania* viability, we employed “genetic metabolite complementation.” First, the trypanosome *de novo* pathway enzymes GDP-mannose dehydratase (GMD) and GDP-fucose synthetase (GMER) were expressed ectopically; from these cells, the Δ*fkp40*^−^/Δ*afkp80*^−^ double mutant was now readily obtained. As expected, the Δ*fkp40*^−^/Δ*afkp80*^−^/+*TbGMD-GMER* line lacked the capacity to generate GDP-Ara*p*, while synthesizing abundant GDP-fucose. These results establish a requirement for GDP-fucose for *L. major* viability and predict the existence of an essential fucoconjugate(s).

## Introduction

*Leishmania* undergoes dramatic changes during its infectious cycle, alternating between a flagellated extracellular promastigote in the midgut of phlebotomine sand flies and an intracellular amastigote residing within macrophages of the mammalian host (1). Upon ingestion of a blood meal by the sand fly vector, *Leishmania* parasites first need to attach to the midgut to avoid excretion and undergo a period of replication and development, after which these procyclic promastigotes differentiate to unbound infective metacyclic promastigotes, adapted for transmission to mammals (2). Lipophosphoglycan (LPG),^3^ the most abundant glycoconjugate on the surface of promastigotes, has been implicated as an adhesion molecule required for midgut attachment in several *Leishmania* species (3).

LPG shows well-defined structural polymorphisms among different *Leishmania* species, with a conserved heptasaccharide core joined to a 1-*O*-alkyl-2-lysophosphatidylinositol anchor. Attached to this glycan core, a long polymer of 15–30 disaccharide phosphate repeating units ([6Gal(β1,4)Man(α1)-PO_4_], also termed phosphoglycan or PG repeats) extends linearly, terminating in a neutral capping oligosaccharide. Importantly, the structure of LPG is developmentally regulated by varying the capping oligosaccharide, the number of PG repeating units, and side chain modifications to the PG repeats in a way characteristic of each *Leishmania* species and critical for interactions with sand fly vector and survival following transmission into the mammalian host (3). One extensively studied pairing involves the interactions of *Leishmania major* strain Friedlin V1 (LmFV1) with its natural host *Phlebotomus papatasi* (4, 5). Procyclic promastigotes express LPG with PG repeats modified by β1, 3-Gal side chain residues mediating binding to the midgut lectin PpGalec (6), as demonstrated by biochemical and genetic studies of LPG and side-chain galactosylation-deficient mutants (7). Upon differentiation to infective metacyclics, LmFV1 synthesizes an LPG in which the side-chain Gal residues are capped with α1,2-d-arapyranose (d-Ara*p*), which is not recognized by midgut lectin and thus permits parasite disengagement in preparation for transmission (8–10).

**Figure 1.**
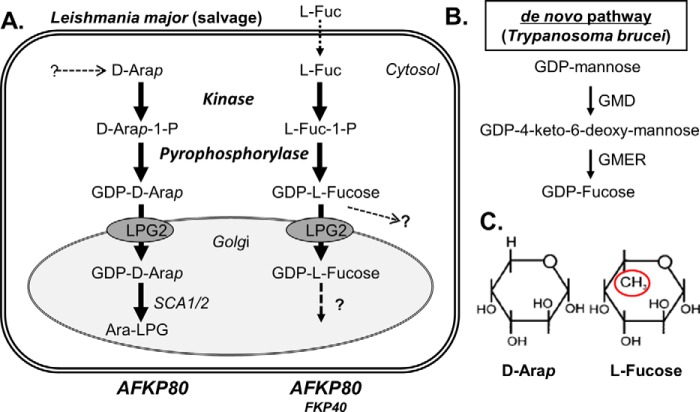
**d-Ara*p* and l-fucose pathways in *Leishmania* and trypanosomes.** *A*, *L. major*. Known metabolic steps (referenced in this paper or established by this work) are depicted with *solid arrows*. d-Ara*p* is known to be synthesized *de novo*, but the steps have not been determined as yet; both d-Ara*p* and l-fucose can be taken up, but the specific carrier(s) has not been established. The presence of fucosyltransferase acting in various cellular compartments is shown in this work, but the specific enzymes and product conjugates remain to be determined. The salvage pathway to GDP-fucose is absent in *T. brucei* but present in *T. cruzi* and humans. *B*, the two enzymes comprising the *de novo* pathway for synthesis of GDP-fucose from GDP-mannose. This pathway is absent in *Leishmania* but present in *T. brucei* as well as *T. cruzi* and humans. *C*, the structures of d-arapyranose and l-fucose. The difference between them is *circled*.

Arabinose exists naturally in both pyranose and furanose conformations and d- and l-configurations. The most abundant form of arabinose is l-arabinose, which is present in the arabinogalactans of plants. d-arabinofuranose (d-Ara*f*) is found mainly in the arabinomannans, arabinogalactans, lipoarabinomannans, and mycolylarabinogalactan-peptidogalactans of mycobacterial cell walls. However, d-Ara*p* is a rare sugar, occurring in several cell surface glycoconjugate structures from certain trypanosomatid parasites: *L. major* and *Leishmania donovani* (11, 12), *Crithidia fasciculata* (13), and *Endotrypanum* spp. (14, 15). In trypanosomatids, d-Ara*p* is synthesized *de novo* by an as yet uncharacterized pathway or taken up from the media and ultimately is converted to the active form GDP-Ara*p* (16). GDP-Ara*p* is then transported into the parasite's Golgi apparatus through the activity of nucleotide sugar transporter LPG2 (17, 18), where it then serves as the donor for arabinosylation of side-chain-galactosylated LPG by the arabinosyltransferases encoded by *SCA1/2* (19, 20). Our knowledge of this pathway is summarized in Fig. 1*A*.

Recently, we identified two closely related *L. major* genes (*LmjF16.0440* and *LmjF16.0480*) showing similarity to a bifunctional kinase-phosphorylase mediating the salvage of fucose to GDP-fucose in *Bacteroides* (21–23). Because d-Ara*p* is structurally similar to l-fucose (Fig. 1*C*), we asked whether the *Leishmani*a enzymes could synthesize GDP-Ara*p*. Studies of the two enzymes expressed from *Escherichia coli* showed that only LmjF16.0480 was able to convert d-Ara through both steps to GDP-Ara*p*. LmjF16.0440 showed strong d-Ara*p* kinase activity but only trace pyrophosphorylase activity. This was attributable to a 3-amino acid difference in the N-terminal pyrophosphorylase enzymatic domain, because the two proteins are otherwise identical (22, 23).^4^ In contrast, when assayed with l-fucose, both *Leishmania* proteins yielded GDP-fucose, although the *V*_max_ for the phosphorylase activity with fucose 1-phosphate of LmjF16.0480 was about 10-fold greater than for LmjF16.0440 (22). Based on their enzymatic activities and their ultimate metabolic role in providing activated GDP-sugar substrates, *LmjF16.0480* was named *AFKP80* (arabino/fucokinase/pyrophosphorylase), whereas *LmjF16.0440* was termed *FKP40* (fucokinase/pyrophosphorylase) (22, 23).^4^ When referred to collectively, the term *A/FKP* can be used.

Although the nature and roles of d-Ara*p*-bearing glycoconjugates are well-known in *L. major*, fucose has never been directly identified for any glycoconjugate isolated from this species. However, there are scattered reports of fucosylation in other *Leishmania* species. The fucosylation of four proteins was inferred from mass spectrometry and proteomics in *L. donovani* (24), and this species synthesizes several complex mannose-fucose-bearing antigens (25). When expressed heterologously in *L. tarentolae*, human erythropoietin was found to bear a fucosylated biantennary *N*-glycan (26). The potential for fucosylation in *L. major* became apparent with the discovery that this species showed significant levels of GDP-fucose (27). Because *L. major* lacks the genes encoding the *de novo* biosynthetic pathway from GDP-mannose to GDP-fucose (GDP-mannose 4,6-dehydratase (GMD) and GDP-fucose synthetase, also known as GDP-4-dehydro-6-deoxy-d-mannose epimerase/reductase (GMER) (28)), GDP-fucose presumably arises via salvage of Fuc through A/FKPs. Fucose may be acquired following digestion of fucosylated molecules abundant in the host or from medium serum supplements. *Trypanosoma brucei* lacks A/FKPs and an active salvage pathway, and GDP-fuc synthesis by the *de novo* pathway is essential (29).

To dissect the potentially overlapping roles of *AFKP80* and *FKP40 in vivo* and the relative contributions of d-Ara*p* and fucose to *Leishmania* biology, we focused on the role of A/FKPs through the generation of null mutants. Surprisingly, whereas we could generate Δ*fkp40*^−^ and Δ*afkp80*^−^ single mutants, we were able to generate Δ*fkp40*^−^/Δ*afkp80*^−^ double mutant only in the presence of ectopically expressed *AFKP80.* Importantly, rescue was also obtained following expression of the GDP-fucose *de novo* pathway genes *GMD* and *GMER*, thereby establishing that GDP-fucose is required for the survival of *L. major*.

## Results

### Generation of Δfkp40^−^ or Δafkp80^−^ null mutants

**Figure 2.**
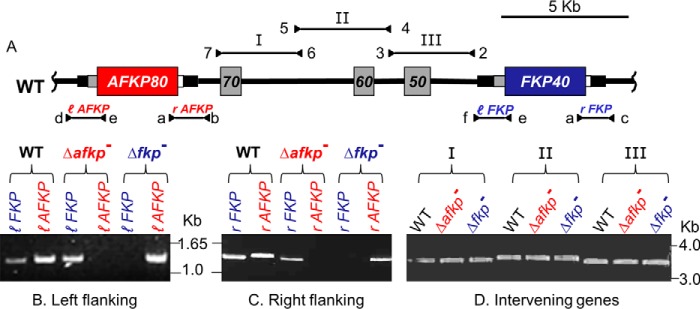
**Generation of *L. major* Δ*afkp80*^−^ and Δ*fkp40*^−^ null mutants singly.** *A*, map of the *A/FKP* locus. The ORFs for *AFKP80 (LmjF16.0480) and FKP40 (LmjF16.0440*) are shown in *red* and *blue*, respectively. The ORFs for the three intervening genes *LmjF16.0470* (hypothetical protein), *LmjF16.0460* (60S ribosomal protein L21), and *LmjF16.0450* (hypothetical protein) are shown in *gray* (*labeled 70*, *60*, and *50*). *Narrow bars* flanking *A/FKP* ORFs depict conserved flanking regions shared by the two genes (*gray*, 5′; *white*, 3′) or the boundaries of the targeting fragments used for gene replacement (*black*). The locations of PCR primers and predicted fragments described in *B–D* are shown *above* or *below* the central map. For clarity, the labels for *AFKP80* or *FKP40*-specific products have been *colored red* or *blue*, respectively; similarly, *l* or *r* designate PCR products probing the left (5′) or right (3′) flanking regions, respectively. Primer sequences can be found in supplemental Table S1. The analysis of the marker replacement alleles is shown in supplemental Fig. S1. *B*, PCR analysis of left (5′) flanking regions of *FKP40* and *AFKP80* in WT, Δ*fkp40*^−^, and Δ*afkp80*^−^. These PCRs establish the presence or loss of either or both of the *A/FKP* genes. Primer e (SMB2453) is common for both *A/FKP* genes, primer d (SMB 2830) is specific for the *AFKP80*, and primer f (SMB2783) is specific for *FKP40. C*, PCR analysis of right (3′) flanking regions of *FKP40* and *AFKP80* in WT, Δ*fkp40*^−^, and Δ*afkp80*^−^. These PCRs establish the presence or loss of *A/FKP* genes as expected. Primer a (SMB2451) is common for *A/FKP*, primer b (SMB2793) is specific for *AFKP80*, and primer c (SMB2784) is specific for *FKP40. D*, PCR analysis of intervening genes in WT, Δ*fkp40*^−^, and Δ*afkp80*^−^. These PCRs establish that the intervening genes are maintained intact as expected. Primers 2 + 3 (SMB3522, 3) amplify regions including the *LmF16.0450* ORF, primers 4 + 5 (SMB3524, 5) amplify the region including LmjF16.0460 ORF, and primers 6 + 7 (SMB3526, 7) amplify the region encompassing the *LmjF16.0470* ORF.

We created null mutants of the *A/FKP* genes individually by homologous gene replacement of each ORF. Because the *Leishmania* genome is predominantly diploid, albeit with occasional aneuploidy (30–33), typically two rounds of gene targeting are required to generate null mutants. Analysis of the *L. major* genome (34) revealed that *AFKP80* and *FKP40* are located on *L. major* chromosome 16, separated by about 12 kb of DNA encoding at least three unrelated genes: *LmjF16.0470* (hypothetical protein), *LmjF16.0460* (60S ribosomal protein L21), and *LmjF16.0450* (hypothetical protein; Fig. 2*A*; labeled *70*, *60*, and *50*). This interspersed arrangement posed additional challenges for gene targeting, further complicated by the sequence similarity between the two *A/FKP* genes (99.9% identity), extending outside of their coding regions 540 nt on the 5′ side and 110 nt on the 3′ side of the two *A/FKP* ORFs (Fig. 2*A*). Reasoning that, as in other organisms, the specificity of homologous recombination is directed by the sequence of the invading linear DNA termini, we used targeting sequences that extended beyond the flanking homology into unique regions (Fig. 2*A*).

For *AFKP80*, we designed constructs that precisely replaced the *AFKP80* ORF with ORFs conferring hygromycin (*HYG*) or puromycin (*PAC*) resistance, flanked by ∼1 kb of 5′ and 3′ *AFKP80* sequence (Fig. 2*A* and supplemental Fig. S1). Successive transfections yielded numerous clonal lines at typical frequencies for *Leishmania* (∼10 clones/μg of DNA). Loss of the *AFKP80* ORF with retention of *FKP40* in the presumptive Δ*afkp80*^−^ mutants was demonstrated by PCR using a common *A/FKP* ORF primer in combination with flanking primers specific for the right or left flanking regions of the *AFK80* or *FKP40* ORFs (Fig. 2*A*). Whereas control WT parasites showed both the expected *AFKP80* and *FKP40* amplicons (Fig. 2 (*B* and *C*), *lanes labeled WT*), the Δ*afkp80*^−^ mutant showed loss of the *AFKP80* but not *FKP40* product (Fig. 2 (*B* and *C*), *lanes labeled* Δ*afkp*^−^). The planned replacement of *AFKP80* was also confirmed by PCR using primer pairs with one located outside of the targeting fragment to either the 5′ or 3′ side, partnered with *HYG*- or *PAC*-specific primers (supplemental Fig. S1).

Similar constructs were made replacing the *FKP40* ORF with markers conferring blasticidin (*BSD*) or nourseothricin (*SAT*) resistance. Loss of the *FKP40* ORF with retention of the A*FKP80* ORF in the presumptive Δ*fkp40*^−^ mutants was demonstrated by PCR showing loss of the *FKP40* but not *AFKP80* product (Fig. 2 (*B* and *C*), *lanes labeled* Δ*fkp*^−^). Furthermore, generation of the planned replacement of *FKP40* was established by PCR using primer pairs, with one located outside of the targeting fragment to either the 5′ or 3′ side, partnered with *BSD*- or *SAT*-specific primers (supplemental Fig. S1).

As an additional control, we confirmed that the three ORFs separating *AFKP80* and *FKP40* were intact. PCRs covering all of the three ORFs and their flanking regions were performed; for both mutants, the PCR products of the proper size were obtained (Fig. 2*D*, *lanes labeled I*, *II*, and *III*).

The Δ*afkp80*^−^ and Δ*fkp40*^−^ mutants showed no obvious morphological change and grew at similar rates and to similar stationary phase cell density as WT in the culture (∼5 × 10^7^ cells/ml; data not shown). To further control the nonspecific effects arising from the disruption of *FKP40* and *AFKP80*, we restored the expression of *FKP40* and *AFKP80* in each mutant, respectively. These complemented lines are referred to subsequently as Δ*afkp80*^−^/+*AFKP80* and Δ*fkp40*^−^/+*FKP40*.

### Enzymatic activities of afkp80 and fkp40^−^ mutants

**Figure 3.**
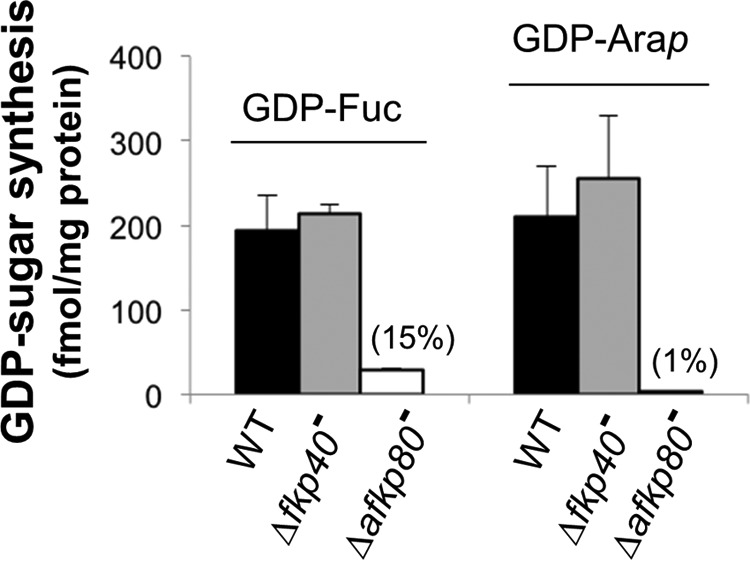
**GDP-fucose and GDP-Ara*p* synthesis in cytosolic extracts from WT and *A/FKP* mutant *Leishmania*.** Cytosolic extracts from WT, Δ*fkp40*^−^, and Δ*afkp80*^−^ were incubated with [^3^H]fucose (*left series*) or [^3^H]d-Ara*p* (*right series*) and the production of radiolabeled GDP-fucose or GDP-Ara*p* was determined as described under “Experimental procedures.” *Black bars*, WT; *gray bars*, Δ*fkp40*^−^; *white bars*, **Δ***afkp80*^−^. The average and S.D. from three independent experiments are shown, calculated using Microsoft Excel 2010. For **Δ***afkp80*^−^, the *numbers* in *parenthesis* were calculated relative to WT.

We prepared cell extracts and evaluated their ability to support the synthesis of GDP-fucose or GDP-Ara*p* from radiolabeled l-fucose or d-Ara*p in vitro*. In WT parasite extracts, comparable activity was seen with either l-fucose or d-Ara*p* (Fig. 3, *black bars*). Thus, *Leishmania* parasites express a fucose salvage pathway with activity comparable with that of d-Ara*p*.

GDP-Ara*p* synthesis was unaffected in extracts from the Δ*fkp40*^−^ mutant, as expected because the recombinant FKP40 enzyme is unable to synthesize GDP-Ara*p*. Consistent with the ability of recombinant AFKP80 to synthesize GDP-Ara*p in vitro*, ablation of this enzyme in the Δ*afkp80*^−^ mutant reduced GDP-Ara*p* synthesis to trace levels (∼1% of WT; Fig. 3). We suspect that this reflects a residual ability of FKP40 to synthesize GDP-Ara*p* that was not measurable in studies of the recombinant enzyme.

GDP-fucose synthesis was not significantly affected in extracts from the Δ*fkp40*^−^ mutant but was reduced to 15% of WT levels in the Δa*fkp80*^−^ (Fig. 3, *gray versus white bars*). Importantly, these data provide evidence that both enzymes mediate GDP-fucose salvage, with the contribution of AFKP80 (present in Δ*fkp40*^−^) being greater than that of FKP40 (present in Δa*fkp80*^−^). Quantitatively, these data are consistent with the kinetic properties of the recombinant enzymes, which were similar other than for the pyrophosphorylase reaction leading to GTP-fucose synthesis from fucose 1-phosphate, where the *V*_max_ was about 10 times greater for AFKP80 than FKP40. Although the close similarity of the two proteins precluded determination of their relative expression, assuming the proteins are expressed at similar levels, the differences in *V*_max_ would predict 91% *versus* 9% activity in the Δ*fkp40*^−^ *versus* Δ*afkp80*^−^ mutants relative to WT, in reasonable agreement with our data (Fig. 3).

Collectively, our findings establish that *in vivo*, *AFKP80* encodes the dominant activity, contributing as expected all of the GDP-Ara*p* synthetic activity and the majority of total cellular GDP-fucose activity.

### Surface arabinosylation is greatly reduced in Δafkp80^−^ but not in Δfkp40^−^ single mutants

**Figure 4.**
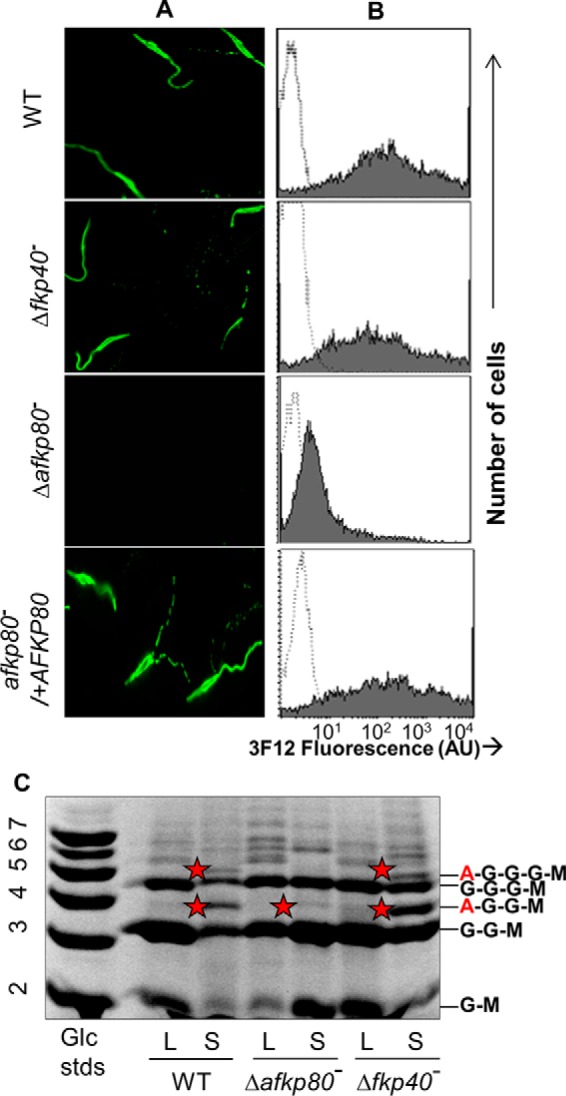
**Surface LPG arabinosylation is greatly reduced in Δ*afkp80*^−^ but is unaffected in Δ*fkp40*^−^**. *A*, indirect immunofluorescence microscopy of fixed promastigote in stationary phase with monoclonal antibody 3F12, specific for d-arabinosylated PG repeating units. The complemented mutant line Δ*afkp80*^−^/+AFKP80 is also shown. *B*, flow cytometry of 3F12 binding (*shaded*); controls omitting 3F12 antibody are shown (*open*). 10,000 cells were counted for each cell line. *AU*, arbitrary fluorescent units. *C*, FACE analysis of *L. major* LPG repeats in log phase (*L*) and stationary phase (*S*). A *red star* denotes repeats containing d-Ara*p*. LPG side chain structures corresponding to each major band are noted on the *right* (Ara*p* (*A*), Gal (*G*), and Man (*M*)). *Lane 1*, glucose oligomer standards (*G2–G7*).

We examined the functional consequences of the single *A/FKP* ablations by assessing the reactivity of the mutants with monoclonal antibody 3F12, which recognizes arabinosylated LPG, specifically the Ara*p*(α1,2)Gal(β1,3)[6Gal(β1,4)Man(α1)-PO_4_] phosphoglycan repeating units (35, 36). Parasites were examined in both logarithmic and stationary phase, a time when metacyclogenesis accompanied by up-regulation of LPG arabinosylation occurs (19). As expected, all log phase parasites showed little reactivity with 3F12 (data not shown). In stationary phase, WT and Δ*fkp40*^−^ mutant parasites showed strong reactivity with 3F12, whereas Δ*afkp80*^−^ parasites showed little reactivity (Fig. 4*A*). Importantly, 3F12 reactivity was restored following introduction of *AFKP80* expression in the Δ*afkp80*^−^/+*AFKP80* “add-back” control (Fig. 4*A*).

These findings were supported by quantitative flow cytometry, again establishing similarly strong 3F12 reactivity in stationary phase WT, Δ*fkp40*^−^, and Δ*afkp80*^−^/+*AFKP80* lines, but with a significant decrease in 3F12 binding to Δ*afkp80*^−^ (Fig. 4*B*). Importantly, these results are exactly those predicted from the enzymatic assays. Although not evident by immunofluorescence, flow cytometry clearly showed a small increase in 3F12 staining (5–6 FU *versus* 2 FU), in keeping with the residual AKP activity in the Δ*afkp80*^−^ mutant (∼1%; Fig. 3). Thus, *in vivo* AFKP80 is dominant, contributing the majority of total cellular GDP-fucose and GDP-Ara*p* synthetic activity.

### LPG arabinosylation is greatly reduced in Δafkp80 but not in Δfkp40

LPG was purified, and the structure of the PG repeating units was assessed by glyco-FACE (fluorophore-assisted carbohydrate electrophoresis). All lines showed similar patterns in log phase, expressing primarily Gal- or Gal-Gal-modified PG repeat units ([Gal-Man-P]^−^), as expected (Gal-Gal-Man-P or Gal-Gal-Gal-Man-P; Fig. 4*C*, *lanes labeled L*). In stationary phase, both WT and Δ*fkp40*^−^ showed a decrease in the synthesis of the Gal-terminated PG repeats and the appearance of Ara*p* capped PG repeats (Ara*p*-Gal-Gal-Man-P or Ara-Gal-Gal-Gal-Man-P; Fig. 4*C*, *lanes labeled* with *S*). In contrast, Ara*p*-capped PG repeat units were barely detectable in stationary phase Δ*afkp80*^−^ parasites (Fig. 4*C*). Western blot analysis of log and stationary WT and Δ*afkp80*^−^ parasites with monoclonal antibody WIC79.3, which preferentially detects the PG repeat units with side chains terminating in Gal (36, 37), showed that the sizes of the LPG were unaltered, with a slightly larger size in stationary phase, consistent with the known increase in PG repeating units in this phase (supplemental Fig. S2). These studies establish a dramatic loss of the Ara*p* modification of LPG in the Δ*afkp80*^−^ but not the Δ*fkp40*^−^ mutant.

### Failure to generate a Δfkp40^−^/Δafkp80 double mutant by “classic” sequential replacements

We reasoned that the properties of an *A/FKP* double mutant could provide information about potential roles of the FKP activities encoded by these genes. The Δ*afkp80*^−^ mutant above was the starting cell line to inactivate *FKP40*, using the same constructs used successfully to create Δ*fkp40*^−^. The *FKP40*::*BSD* replacement fragment was first transfected into Δ*afkp80*^−^, yielding clonal lines without difficulty that were typed as Δ*afkp80*^−^/Δ*fkp40*::*BSD*/*FKP40*, as expected. These “3/4 *A/FKP* replacement” transfectants grew normally. One line was then transfected with the targeting fragment *FKP40*::*SAT* and plated on semisolid medium containing all four marker-selective drugs. Whereas transfectants were obtained without difficulty, PCR tests of over 50 transfectants from three independent experiments showed that all contained successful *FKP40* replacements with the *BSD*- and *SAT*-selectable markers as well as retaining a copy of *FKP40* (supplemental Fig. S3). A similar strategy attempting to inactivate *AFKP80* in the Δ*fkp40*^−^ mutant yielded similar results (data not shown).

The finding of seemingly successful “double replacements” accompanied by retention of the WT gene has been reported previously in attempts to target other essential genes in *Leishmania* and shown to arise from generation of aneuploid or tetraploid parasites (31, 38), although the strength of this conclusion has been mitigated by the appreciation of widespread aneuploidy within most laboratory strains of *Leishmania* (33). Measurements of DNA content by flow cytometry after propidium iodide staining of the quadruply drug-resistant “double transfectants” showed a pattern similar to that of WT (data not shown), suggesting that these lines were likely to be aneuploid.

### Plasmid segregation tests establish the essentiality of A/FKPs

**Figure 5.**
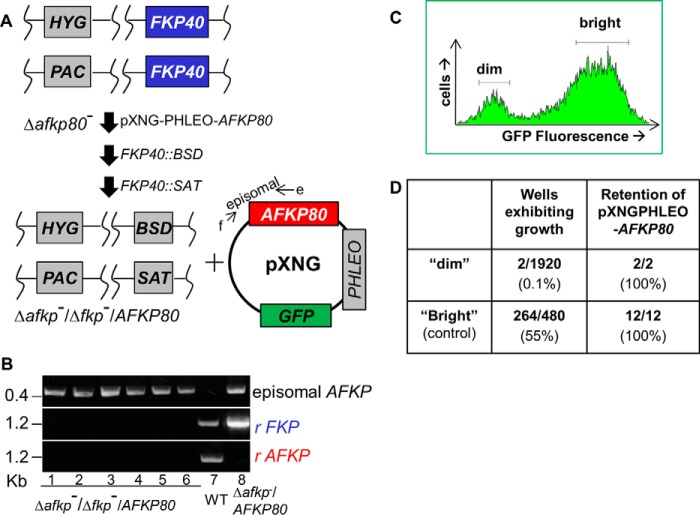
**Plasmid segregational test of *A/FKP* essentiality.** *A*, workflow for deletion of *A/FKP* genes in the presence of ectopic *AFKP80*. First, a pXNG-*AFKP80* episomal expression construct was transfected into the Δ*afkp80*^−^ mutant, yielding Δ*afkp80*^−^/+pXNG-*AFKP80*. Following confirmatory PCR tests, one clonal line was sequentially transfected with *FKP40*::*BSD* and *FKP40*::*SAT* targeting fragments, resulting in loss of *FKP40. B*, PCR confirmation of loss of chromosomal *FKP40* and *AFKP80* using primers as described in Fig. 2 and the presence of episomal *AFKP80* confirmed by PCR using primer f (specific in pXNG vector; SMB 3176) paired with the *AFKP80* primer e (SMB2453). *C*, plasmid segregational tests of *A/FKP* essentiality. Δ*afkp80*^−^/Δ*fkp40*^−^*/*+pXNG-*AFKP80* was grown for 24 h in the absence of phleomycin and analyzed by GFP flow cytometry. For subsequent quantitation and/or sorting, weakly fluorescent (dim) and fluorescent (bright) parasites were defined as shown in the figure. *D*, single cells from both GFP dim and bright populations gated (Fig. 5*C*) were sorted into 96-well plates containing M199 medium; the numbers sorted and their growth and properties are shown. The two survivors from the “dim” and 12 of the 12 from the “bright” population sort were tested for retention of pXNGPHLE-*AFKP80* by growth in medium containing phleomycin.

Recently, we introduced a plasmid segregational approach as a more stringent, controlled test of gene essentiality (38). First, we inserted *AFKP80* into the pXNGPHLEO vector, which expresses the GFP+ reporter gene, and transfected this into the Δ*afkp80*^−^ mutant (Δ*afkp80*^−^/pXNGPHLEO-*AFKP80*). The genotype was confirmed by PCR tests, and these lines showed normal arabinosylation, as expected (not shown). Then we performed successive transfections with the *FKP40*::BSD and *FKP40*::*SAT* replacement constructs used successfully to inactivate *FKP40* above (Fig. 5*A*). In contrast to the “classic” four replacement attempts without ectopically expressed *AFKP80*, each transfectant showed successful deletion of the chromosomal copy of *FKP40* and *AFKP80* (Fig. 5*B*). PCR tests confirmed that the marker replacements occurred as planned and that the lines maintained the three intervening genes (as described in Fig. 2*A* and supplemental Fig. S1; data not shown). Thus, in the presence of ectopically expressed *AFKP80*, all chromosomal *A/FKP* genes could be successfully eliminated, generating Δ*afkp80*^−^/Δ*fkp40*^−^/+pXNGPHLEO-*AFKP80*.

To perform plasmid segregation tests, parasites were grown briefly (24 h) in the absence of phleomycin (selective for the *PHLEO* marker of pXNG) (38). The parasites were then analyzed for GFP expression by flow cytometry, as a measure of pXNG copy number. Two populations were revealed: a large population of “bright” cells showing strong fluorescence bearing high copy numbers of pXNG-*AFKP80* (>200 FU; Fig. 5*C*) and a smaller population of “dim” cells, exhibiting control/background fluorescence levels (2–20 FU; Fig. 5*C*), presumably lacking pXNG-*AFKP80* completely or bearing only a few copies.

Fluorescence-activated cell sorting was then used to recover single cells into individual wells of a 96-well microtiter plate, containing M199 medium without phleomycin. For the bright cell population, 264 of 480 cells inoculated with single bright parasites grew out (55%), representing the “cloning/plating” efficiency of cells subjected to this protocol. In contrast, growth was seen in only 2 of 1920 of the dim parasites tested similarly (0.1%; Fig. 5*D*). The two survivors still retained pXNG-*AFKP80*, as judged by their ability to grow out in the presence of phleomycin and expression of GFP (data not shown), and were thus likely to arise by imperfect sorting or other technical factors, as seen previously (38). After correction for the plating/cloning efficiency, we estimated that parasites lacking both *A/FKP* genes were not obtained from ∼1056 cells tested, thereby extending the stringency of this conclusion beyond that possible by the classic approach (∼50 cells tested).

### Rescue of a Δafkp80^−^/Δfkp40^−^ double mutant by introduction of a de novo GDP- fucose pathway

We hypothesized that inviability of the Δ*afkp80*^−^/Δ*fkp40*^−^ mutant could arise if GDP-fucose was unexpectedly essential in *Leishmania*. One way to test this was by “genetic metabolic complementation” (38), through introduction of an alternative source of GDP-fucose. Because *Leishmania* normally lack the *de novo* pathway (Fig. 1*A*), we chose to introduce that from *T. brucei*, comprising three enzymatic activities encoded by two proteins (GMD and GMER) that have proven amenable to genetic manipulation (29).

First, we inserted the two genes required (*TbGMD* and *TbGMER*) (29) into the bicistronic expression vector pIR1PHLEO, yielding pIR1PHLEO-*TbGMD-GMER* (see “Experimental procedures”). This construct was introduced into the penultimate “3/4” replacement line described earlier, yielding Δ*afkp80*^−^/Δ*fkp40*::*BSD*/*FKP40*/+*TbGMD-TbGMER*. In this experiment, the pIR vectors were transfected without linearization, which yields circular episomal transfectants; these overexpress passenger molecules, but not to the same extent as when inserted into the rRNA locus (39).

**Figure 6.**
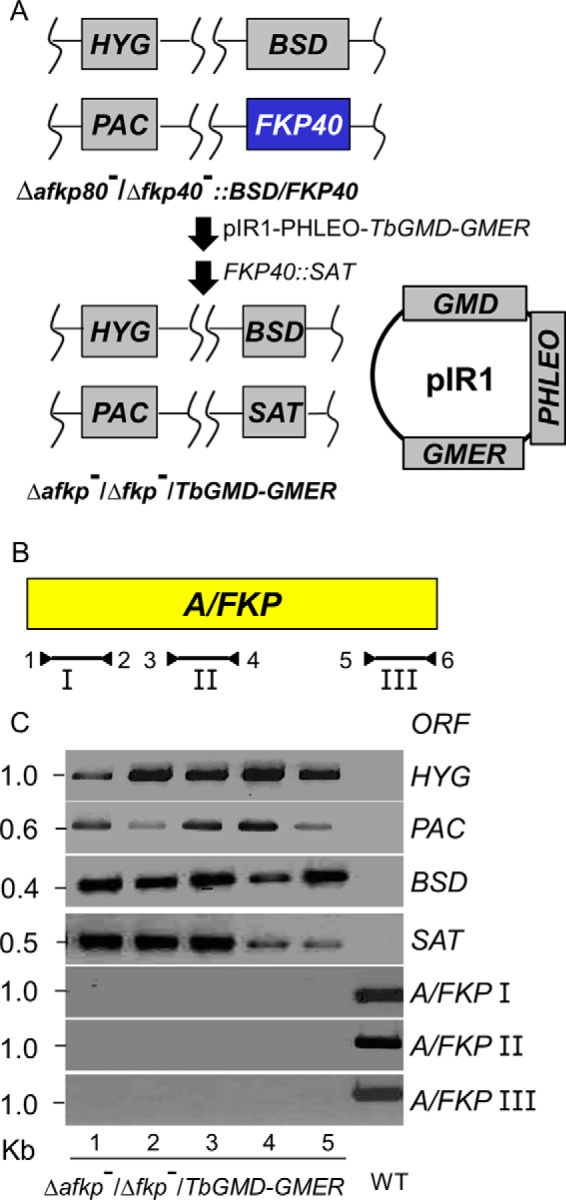
**Genetic metabolite complementation permits the recovery of Δ*afkp80*^−^/Δ*fkp40*^−^ double null mutants.** *A*, workflow for the deletion of chromosomal *FKP40* in the Δ*afkp80*^−^ mutant, in the presence of the *de novo* GDP-fucose pathway encoded by *TbGMD and TbGMER*. First, pIR1PHLEO-*TbGMD-GMER* was transfected into the intermediate line Δ*afkp80*^−^/Δ*fkp40*::BSD/*FKP40* depicted in Fig. 6*A* (this line can be considered a “3/4” *A/FKP* replacement). After confirmatory PCR tests, this line was then transfected with the *FKP40*::*SAT* targeting fragment to replace the last copy of *FKP40,* yielding the line termed Δ*afkp80*^−^/Δ*fkp40*^−^/+*TbGMD-TbGMER. B*, scheme of primers used to confirm loss of all *A/FKP* alleles. Primer 1, SMB2828; primer 2, SMB3544; primer 3, SMB2446; primer 4, SMB3752; primer 5, SMB 2450; primer 6, SMB2829. *C*, PCR tests showing loss of all four *A/FKP* alleles in Δ*afkp80*^−^/Δ*fkp40*^−^/+*TbGMD-TbGMER* and the presence of all four selective markers. *Lines 1–5* represent five independent lines with four replacements removing all *A/FKP* genes. Not shown are PCR tests confirming the presence of the pIR1PHLEO-*TbGMD-TbGMER*, whose presence is confirmed by GDP-fucose synthesis (Table 1).

Finally, the remaining *FKP*40 allele was removed by transfection with the *FKP*::*SAT* targeting fragment (Fig. 6). In contrast to the studies above with WT lines lacking a functional *de novo* GDP-fucose pathway, now all transfectants (Δ*afkp80*^−^/Δ*afkp40*^−^/+*TbGMD-TbGMER*) showed loss of all *A/FKP* alleles (Fig. 6). PCR tests showed that these lines retained all chromosomal markers as planned, as well as the three intervening ORFs (similar to those shown in Fig. 2*A*, supplemental Fig. S1, or Fig. 6*C*, or data not shown). Thus, expression of a functional *de novo* GDP-fucose synthetic pathway is able to bypass the absence of all *A/FKP* genes.

### Δafkp80^−^/Δfkp40^−^/+TbGMD-TbGMER lacks GDP-Arap while synthesizing abundant GDP-fucose

Sugar nucleotides were analyzed in WT and Δ*afkp80*^−^/Δ*fkp40*^−^/+*TbGMD-TbGMER* double mutants grown in logarithmic and stationary phase cells, as described previously (27). As expected, no GDP-Ara*p* was detectable in the *A/FKP*-null mutant, whereas WT levels were similar to those reported previously (Table 1) (27). Second, GDP-fucose expression rose tremendously in the episomal *TbGMD/GMER* transfectant *A/FKP* null mutant, increasing 129- or 95-fold in logarithmic or stationary phase cells, respectively (Table 1).

**Table 1:** **Nucleotide-sugar levels in WT and the Δ*afkp80*^−^/Δ*fkp40*^−^*/+TbGMD-TbGMER* mutant *L. major*** The averages ± S.D. from 2–3 experiments with 2–3 replicas each are shown. Asterisks mark results where the mutant significantly differs from WT; *, *p* < 0.05; **, *p* < 0.005 (two-sided *t* test with unequal variance). S.D. and statistical tests were calculated using Microsoft Excel 2010. ND, not detectable.

Nucleotide sugar	Log phase		Stationary phase	
	WT	Δ*afkp80*^−^/ Δ*fkp40*^−^*/ +TbGMD-TbGMER*	WT	Δ*afkp80*^−^/ Δ*fkp40*^−^*/ +TbGMD-TbGMER*
	*pmol/10^7^ cells*		*pmol/10^7^ cells*	
UDP-hexose	12.9 ± 8	12.6 ± 8.6	1.05 ± 0.42	0.26 ± 0.16**
UDP-GlcNAc	23.8 ± 9.2	17.5 ± 13.9	1.94 ± 1.30	0.31 ± 0.24**
GDP-Man	1.07 ± 0.49	0.57 ± 0.72	0.20 ± 0.05	0.20 ± 0.21
GDP-Ara*p*	0.17 ± 0.11	ND	0.21 ± 0.14	ND
GDP-Fuc	0.06 ± 0.11	6.7 ± 5.3*	0.35 ± 0.14	11.4 ± 5.5**

These data established that the *de novo* pathway had been successfully transplanted into *L. major*. Importantly, the great increase in GDP-fucose over that seen in WT was not accompanied by a significant drop in the vital GDP-mannose precursor in either growth phase (which, in fact, rose somewhat in stationary phase; Table 1). This suggests that the parasites were able to autoregulate and compensate for the increased flux through the GDP-mannose synthetic pathway toward GDP-fucose.

Little change was seen in the UDP-GlcNAc or UDP-hexose levels in log phase, although a modest drop to 16 and 22% of WT levels, respectively, was seen in stationary phase (Table 1). Neither change reached statistical significance (*p* < 0.05) in the studies performed. Curiously, perturbations of UDP-glucose and/or galactose synthesis following genetic perturbations of UDP-sugar pyrophosphorylase (*USP*) or UDP-Glc pyrophosphorylase (*UGP*) also led to changes in GDP-fucose levels (40). This suggests the possibility that GDP-fucose and UDP-Gal synthesis may be co-regulated for some reason, perhaps related to the structure of hypothetical fucoconjugates, although this has not been studied further.

### Preliminary evidence for fucoconjugates in L. major

**Figure 7.**
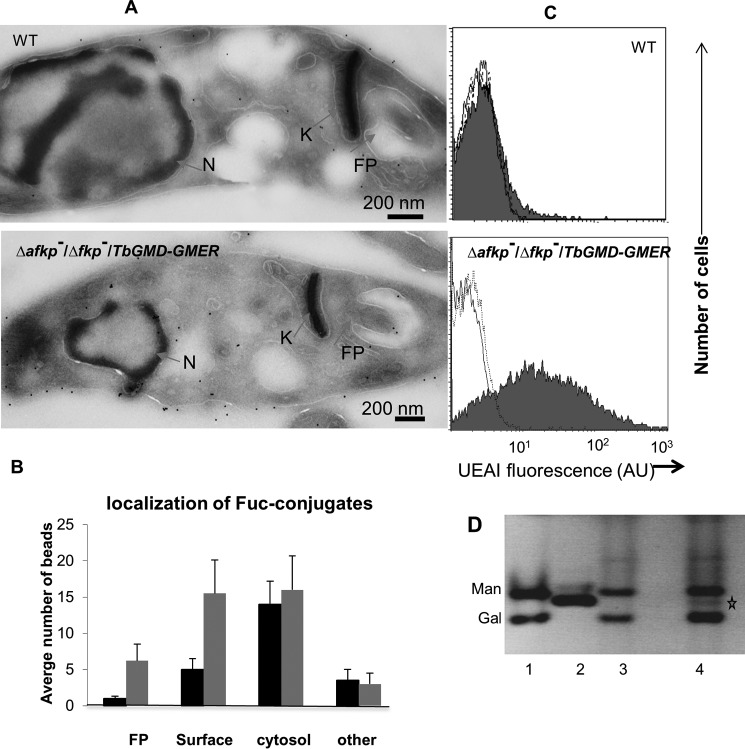
**Evidence for fucosylation in WT and Δ*fkp40*^−^/Δ*afkp80*^−^/+*TbGMD-GMER L. major*.** *A*, binding of biotinylated UEA-I lectin/streptavidin gold particles detected by cryo-EM (see “Experimental procedures”). *FP*, flagellar pocket; *k*, kinetoplast; *N*, nucleus. *B*, quantitation of cellular location of UEA-I-bound gold particles in WT and mutants. Three experiments were performed, counting 10 cells from each line; the averages and S.D. values are shown, calculated using Microsoft Excel 2010. “Other” locations include the mitochondrion, Golgi apparatus, and endoplasmic reticulum. *C*, WT or Δ*afkp80*^−^/Δ*fkp40*^−^/+*TbGMD-TbGMER* parasites were washed with PBS, incubated with fluoresceinated UEA-I lectin (*UEAI*; shaded) or PBS (open), or fluoresceinated UEA-I lectin plus 25 mm fucose (dotted), and then subjected to flow cytometry. Representative experiments from three independent replicas for each line are shown. *D*, glyco-FACE analysis. *Lane 1*, Man + Gal standard; *lane 2*, fucose standard; *lane 3*, WT; *lane 4*, Δ*fkp40*^−^/Δ*afkp80*^−^/+*TbGMD-GMER.* A representative experiment from three independent replicas is shown.

As yet no evidence of fucosylated molecules occurring naturally in *L. major* has been presented, although when provided with high levels in the medium, fucose can be substituted for d-Ara*p* on LPG (41). For preliminary evidence of fucoconjugates, we used cryo-electron microscopy and binding to biotinylated *Ulex europaeus* agglutinin I (UEA-I) lectin, which recognizes terminal α1,2-fucosyl linkages (42) in WT and the Δ*afkp80*^−^/Δ*fkp40*^−^/+*TbGMD-TbGMER* mutant synthesizing elevated GDP-fucose. UEA-I-conjugated gold particles were counted across several cellular compartments in both the mutant and WT parasites (Fig. 7*A*,). Increased numbers of particles were seen only in the flagellar pocket and on the parasite surface in the GDP-fucose-overexpressing TbGMD/GMER transfectant (3–6-fold; Fig. 7*B*). Comparable levels were seen in the cytosol and other compartments, including the mitochondrion, endoplasmic reticulum, and Golgi apparatus (Fig. 7*B*).

The cryo-EM data were confirmed by flow cytometry of non-permeabilized cells allowed to bind the fucose-specific fluoresceinated UEA-I. WT *Leishmania* showed little reactivity (Fig. 7*C*), whereas Δ*afkp80*^−^/Δ*fkp40*^−^/+*TbGMD-TbGMER* showed a significant increase in fluoresceinated UEA-I fucose lectin binding (∼10-fold; Fig. 7*C*, *gray shading*). Importantly, the addition of fucose totally inhibited the reactivity (Fig. 7*C*, *dotted line*).

The *Leishmania* genome predicts at least five candidate arabinosyl/fucosyl transferases, including the two encoded by *SCA1/2* that reside within the secretory pathway, where they normally mediate LPG arabinosylation or fucosylation if cells are provided with high levels of fucose (19, 41). LPG was isolated from WT and Δ*afkp80*^−^/Δ*fkp40*^−^/+*TbGMD-TbGMER,* hydrolyzed, and analyzed by glyco-FACE (see “Experimental procedures”). In these analyses, fucose was undetectable in WT LPG, whereas it was present at about 5–8% the level of mannose in the GDP-fucose overexpresser (which occurs mostly as a single residue within the LPG phosphoglycan repeats) (Fig. 7*D*). This is consistent with the hypothesis that the elevated UEA-I reactivity in the TbGMD/GMER expresser mainly arises from fucosylated LPG. Regardless, our data establish that the high levels of GDP-fucose in the TbGMD/GMER expressers are accessible within the secretory pathway.

### The AFKP80 and FKP40 proteins are located in the cytoplasm

**Figure 8.**
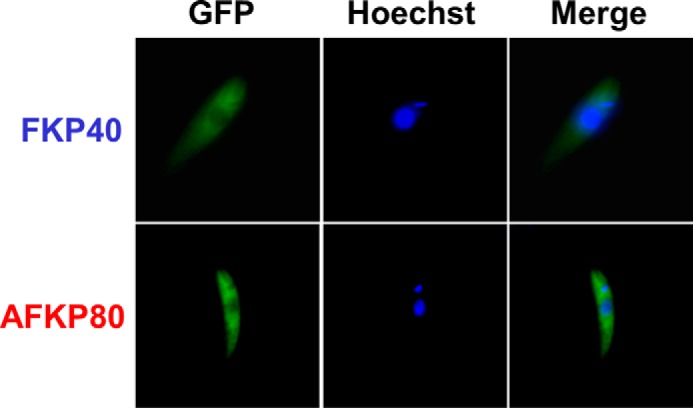
**GFP-tagged *L. major* FKP40 and AFKP80 are localized in the cytoplasm.** Wild-type *L. major* expressing FKP40 or AFKP80 bearing an N-terminal GFP+ tag (WT/+pXG-*GFP-FKP40* or WT/+pXG-*GFP-AFKP80*) were stained with Hoechst 33342 dye and visualized by fluorescence microscopy. *Left panels*, GFP fluorescence; *center panels*, Hoechst fluorescence; *right panels*, merged.

In trypanosomatids, nucleotide sugars are synthesized in the cytosol or glycosome (27, 43) and enter the secretory pathway through the action of nucleotide-sugar transporters (44). GDP-mannose, GDP-fucose, and GDP-Ara*p* have been shown previously to be substrates for the nucleotide-sugar transporter encoded by *LPG2* (Fig. 1*A*) (17, 45, 46). We constructed N-terminal GFP-tagged versions of both *FKP40* and *AFKP80* and introduced them separately into WT parasites. Fluorescence microscopy showed that the GFP-tagged FKP40 and AFKP80 were both distributed throughout the whole-cell WT (Fig. 8), consistent with an absence of predicted cellular targeting signals within these two polypeptides. Δ*afkp80*^−^/+*GFP-AFKP80* transfectants showed full 3F12 (Ara*p*-LPG) reactivity, confirming that the GFP tag did not compromise enzymatic activity (data not shown).

## Discussion

In this work, we used single and double gene knockouts as well as “genetic metabolic complementation” to probe the contributions of the two closely related A/FKP genes to *L. major* metabolism. Previous studies of recombinant enzymes had shown that only AFKP80 could mediate synthesis of GDP-d-Ara*p*, whereas both AFKP80 and FKP40 could mediate synthesis of GDP-fucose (22, 23).^4^ Consistent with these data, ablation of *AFKP80* resulted in reduction of AKP activity to trace levels as well as an 85% decrease in FKP activity (Fig. 3). Correspondingly, LPG arabinosylation was entirely eliminated, suggesting that this is the sole route of GDP-Ara*p* synthesis in the parasite (Fig. 4). Because LPG itself is not essential *in vitro* (47), it was unsurprising that lack of LPG arabinosylation had no effect on cell viability or growth.

In contrast, for *FKP40*, which is able only to mediate the synthesis of GDP-l-fucose, genetic ablation yielded no detectable phenotype. Cells grew normally, with WT morphology, and maintained LPG side chain arabinosylation (Fig. 4). This could not be attributed to a lack of FKP40 expression or activity, because the Δ*afkp80*^−^ mutant, which retains only *FKP40*, showed about 15% of WT levels of GDP-fucose synthesis (Fig. 3), consistent with studies of recombinant enzymes *in vitro*.^4^

Given the paucity of evidence for an important role for l-fucose in *Leishmania* and the nonessential role for d-Ara*p*, we attempted to generate a double *A/FKP* mutant, expecting that this would confirm the lack of a requirement for GDP-fucose or fucosylation. However, we were unable to create the double mutant. Various controls ruled out technical issues, and the double mutant could be obtained in the presence of ectopic *AFKP80* expression (Fig. 5). Importantly, plasmid segregation tests of > 1000 cells expressing ectopic *AFKP80* in a double chromosomal null background showed that it was impossible to generate cells lacking both of the *A/FKP* genes (Fig. 5).

These data raised the possibility that unlike GDP-Ara*p*, GDP-fucose was an essential metabolite in *L. major*. To confirm this hypothesis, we employed the approach of “genetic metabolic complementation” (38), introducing an alternate, “bypass” route of metabolite synthesis to confirm the role of the primary target (provision of GDP-fucose). This was accomplished by introduction of the well-characterized *de novo* GDP-fucose pathway from the closely related protozoan *T. brucei*, which, inverse from *Leishmania*, lacks a salvage pathway (29). Successful expression was shown by the synthesis of high levels of GDP-fucose (Table 1). In the presence of an active *de novo* GDP-fucose pathway, we were readily able to generate chromosomal null mutants lacking any *A/FKP* genes whatsoever (Fig. 6). These data argue strongly that GDP-fucose is an essential metabolite in *Leishmania*.

We exploited the greatly elevated levels of GDP-fucose in transfectants expressing TbGMD/GMER to facilitate visualization of fucoconjugates normally present at undetectably low levels. Increased UEA-I lectin binding signal was seen over the flagellar pocket and cell surface (Fig. 7, *B* and *C*), potentially arising from any or all of the candidate fucosyltransferases, targeted to the secretory pathway. Some evidence of cytoplasmic fucoconjugates also emerged, (Fig. 7, *A–C*). The *Leishmania* genome encodes a candidate homolog of *SKP1* (LmjF.11.1210), a cytosolic protein identified first in *Dictyostelium* and known to bear essential fucosyl modifications in other unicellular microbes (48, 49). We did not see prominent staining of nuclear structures, in contrast to *Toxoplasma gondii*, where fucosylation of nuclear pore complexes may occur (50).

Collectively, our data suggest that *L. major* may synthesize a variety of fucoconjugates, at least one of which must be essential. Whereas LPG can be fucosylated in the presence of high levels of fucose (exogenous or endogenous; Fig. 7*D*) (41), the essential glycoconjugate(s) is unlikely to be LPG, because LPG-null mutants are viable *in vitro* (47). Because *Leishmania* lack the *de novo* fucose pathway and rely entirely on salvage, we speculate that in our studies *in vitro* and probably *in vivo*, the fucose requirement may be satisfied through ingestion and catabolism of fucosylated proteins.

The *de novo* GDP-fucose pathway is also essential in *T. brucei*, where the essential fucoconjugate similarly remains unknown (29). In contrast, the *T. cruzi* genome predicts the presence of both the *de novo* and salvage pathways. *T. cruzi* synthesizes a complex fucose-containing surface glycan attached to gp72 (51–53), ablation of which results in flagellar detachment (54). Although the *T. brucei* gp72 ortholog is not known to be fucosylated, its ablation leads to death preceded by flagellar detachment and blockage of cell division (55, 56). The *Leishmania* genome predicts at least one gp72 ortholog (LmjF10.0630), whose role(s) and/or modifications have not been studied. GDP-fucose synthesis and fucoconjugates have also been described in apicomplexan parasites. In *T. gondii*, the *de novo* GDP-fucose pathway appears to be essential, possibly due to a role in nuclear pore complex modification (50). Protein *O*-fucosylation of the surface CSP and TRAP sporozoite proteins of the malaria parasite *Plasmodium falciparum* has been described (57), although the *de novo* pathway for GDP-fucose synthesis does not appear to be essential (58, 59).

Formally, the ability of the GDP-fucose *de novo* pathway to rescue pan-*A/FKP* mutants might not imply that GDP-fucose itself is essential. Potentially, an unknown downstream metabolite could be responsible, or perhaps GDP-Ara*p* itself is essential, and GDP-fucose rescues simply by mimicry. We think these scenarios are unlikely to rule out a role for GDP-fucose for the following reasons: 1) GDP-fucose is typically a terminal metabolite, not undergoing further modification; 2) WT *L. major* synthesize GDP-fucose (27); and 3) candidate fucoconjugates have been reported in closely related species (24–26, 60).

Instead, it seems more likely that there remain new, unidentified fucoconjugates to be found. In all probability, these will differ significantly from known parasite glycoconjugates, probably occurring at low levels, yet playing vital role(s) in parasite biology. Our studies now elevate the priority of studies seeking to characterize the fucoconjugate repertoire of *Leishmania*, at least one of which is predicted to play a vital role(s) in parasite biology.

## Experimental procedures

### Leishmania culture and transfection

*L. major* strain Friedlin V1 (MHOM/IL/80/Friedlin) was grown at 26 °C in M199 medium (U.S. Biologicals) containing 10% heat-inactivated fetal bovine serum and other supplements as described (61). *Leishmania* cells were transfected by electroporation using a high-voltage protocol (62). Following transfection, cells were allowed to grow for 16–24 h in M199 medium and then plated on semisolid media containing 1% Nobel agar (Fisher) and appropriate selective drugs (50 μg/ml hygromycin B, 30 μg/ml puromycin, 10 μg/ml blasticidin, 100 μg/ml nourseothricin, 10 μg/ml phleomycin, and/or 10 μg/ml G418). Individual colonies were picked and grown in liquid medium in same drug concentration as used in plates. Clones were maintained in selective medium and then removed from selection for one passage before experiments.

### Flow cytometry and immunofluorescence microscopy

Parasites were washed in PBS and fixed with 4% paraformaldehyde for 10 min at room temperature. For immunofluorescence labeling, cells were immobilized on poly(l-lysine)-coated glass coverslips and blocked with 5% normal goat serum. Fixed parasites were then sequentially incubated for 1 h at room temperature with primary and secondary antibodies diluted in 5% normal goat serum (63). Flow cytometry was performed with a BD Biosciences FACSCalibur system. Monoclonal antibody 3F12 was used at 1:100 dilutions, Fluor 488 goat anti-mouse IgG was used at 1:1000, and fluoresceinated UEA-I was used at 10 μg/ml.

### Targeted gene replacement of L. major FKP40 and AFKP80

Fusion PCR was used to generate replacement constructs. Briefly, the flanking regions and drug resistance cassettes were amplified separately by PCR using primers that produce overlapping ends. An 870-nt 5′ region and a 930-nt 3′ region of *FKP40* were amplified using primer pairs SMB 2664/2665 and SMB 2666/2667 (primer sequences are listed in supplemental Table S1). The ORFs of blasticidin (*BSD*) and nourseothricin (*SAT*) were amplified from pXGBSD (strain B4098) and pXGSAT (strain B2352), respectively, using primers with added linker sequence. A linear DNA *BSD* or *SAT* between 5′- and 3′-flanking regions of *FKP40* was synthesized in a second round of PCR by mixing the purified PCR products of the flanking regions and a drug-resistance cassette as templates using primers SMB 2664/2667 and then inserted into pGEM-T (Promega) to make pGEM-*FKP40-BSD* (strain B5925) and pGEM-*FKP40-SAT* (B5926), respectively. The targeted linear fragments were liberated from pGEM-*FKP40-BSD* and pGEM-*FKP40-SAT* by digestion with BsmI and DraIII, respectively; treated with calf intestinal phosphatase; purified after agarose gel electrophoresis; and transfected into LmFV1. The heterozygous mutant Δ*fkp40*::*BSD*/*FKP40* was obtained by transfecting 5 μg of *FKP40*::*BSD* fragment into WT LmFV1 promastigotes as described previously (62). A second targeting round with *FKP40*::*SAT* resulted in Δ*fkp40*::*BSD*/Δ*fkp40*::*SAT*, referred to as the Δ*fkp40*^−^ mutant. Before study, all lines were passed through mice once by injecting hind footpads of BALB/c mice (Charles River Laboratories, Wilmington, MA) with a large inoculum (1–5 × 10^7^) of stationary-phase parasites and recovering parasites by needle aspiration of footpad regardless of pathogen 4 weeks afterward.

A similar strategy was used to create the Δ*afkp80*^−^ mutant. *AFKP80* allelic replacement constructs were made by inserting ORFs encoding hygromycin B (HYG) or puromycin (PAC) resistance between 930-nt 5′ and 980-nt 3′ *AFKP80* flanking regions, making pGEM-*AFKP80-HYG* (strain B5950) and pGEM-*AFKP80-PAC* (strain B5961). Targeting fragment was liberated from pGEM-*AFKP80-HYG* by NspI and StuI, purified before transfection into LmFV1. The second round replacement was performed by electroporation of *AFKP80*::*PAC* fragment excised from pGEM-*AFKP80-PAC* (strain B5961) by NdeI and KpnI, resulting in Δ*afkp80*::*HYG*/Δ*afkp80*::*PAC*, hereafter referred to as the Δ*afkp80*^−^ mutant. Using the same constructs and similar methods, we attempted unsuccessfully to make a Δ*fkp40*^−^/Δ*afkp80*^−^ double mutant by inactivating *FKP40* in the *afkp80*^−^ mutant or *AFKP80* in the *fkp40*^−^ mutant.

The ORFs of *FKP40* and *AFKP80* were PCR-amplified from LmFV1 genomic DNA using primer pairs SMB 2828/2829 with an XmaI site included. For expression, an optimal translation sequence (CCACC) was added upstream of the ORF starting codon in primer 2828. Because there is only a 3-base difference in the middle of the ORFs between *FKP40* and *AFKP80*, PCR products were a mixture of *FKP40* and *AFKP80*. PCR products were then directly cloned into pGEM-T vector by TA cloning. Some clones were sequenced, sorting out pGEM-*FKP40* (B5988) and pGEM-*AFKP80* (B5989). The ORFs were then liberated with XmaI and inserted in the sense direction of the XmaI expression site of pXG (NEO-B1288), creating pXG-*FKP40* (B5990) and pXG-*AFKP80* (B5992). To restore the expression of *FKP40* or *AFKP80*, *fkp40*^−^ and *afkp80*^−^ mutants were transfected with 5 μg of pXG-*FKP40* and pXG-*AFKP80*, respectively. For simplicity, in this work, the transfectants Δ*fkp40*::*BSD*/Δ*fkp40*::*SAT* [pXG-*FKP40*] are designated Δ*fkp40*^−^/+*FKP40*; similarly, the transfectants Δ*afkp80*::*HYG*/Δ*afkp80*::*PAC* [pXG-*AFKP80*] are designated Δ*afkp80*^−^/+*AFKP80*.

### Subcellular localization of L. major FKP40 and AFKP80

The ORFs of *FKP40* and *AFKP80* were excised from pGEM-*FKP40* (B5988) and pGEM-*AFKP80* (B5989) by digestion with NotI and inserted in the sense orientation of the NotI site of pXG-GFP+2 (B2952), yielding pXG-*GFP-FKP40* (B6250) and pXG-*GFP-AFKP80* (B6251). Both constructs were transfected into WT LmFV1, and clonal lines were obtained and verified. As a control, pXG-*GFP-AFKP80* was also transfected into Δ*afkp80*^−^ mutant. WT/+*GFP-FKP40* and WT/+*GFP-AFKP80*promastigotes were fixed with 0.5% (w/v) paraformaldehyde in PBS and immobilized on coverslips. Hoechst 33342 staining (5 μg/ml in PBS) was performed to visualize nuclear and kinetoplast DNAs.

### Cytosolic fractionation and enzymatic assay

Stationary phase parasites were suspended in 12 ml of lysis buffer (100 mm HEPES, pH 7.4, 50 mm KCl, 10% glycerol, and EDTA-free protease inhibitor mixture) and lysed by nitrogen cavitation (1500–2000 p.s.i., incubated on ice for 30 min). The crude lysate was centrifuged at 100,000 × *g* for 1 h at 4 °C, and the supernatant was used as a source of cytosolic enzymes. The standard assay mix contained 100 μl of the cytosolic fraction, 5 mm final concentration each of ATP, GTP, and MgSO_4_; 1 unit of inorganic pyrophosphatase; and 0.15 μCi of d-[5-^3^H]arabinose (20 Ci/mmol) or l-[6-^3^H]fucose (60 Ci/mmol). Assay mixtures were incubated at 37 °C for 16 h and terminated by boiling for 1 min. Nucleotide sugars were purified by anion-exchange chromatography, as described earlier (64). Briefly, the reaction mixtures were applied to a column (1.5 × 7.5 cm) of DE-52 cellulose and washed with 50 ml of water. The products of the reaction were eluted with a 120-ml gradient (0–250 mm) of (NH_4_)HCO_3_. GDP-Ara*p* and GDP-fucose emerged with 120–150 mm (NH_4_)HCO_3_ and were subjected to scintillation counting.

### Purification and analysis of LPG

LPG was extracted and purified by phenyl-Sepharose chromatography (65). Briefly, exponentially growing (1–2 × 10^6^ cells/ml) or stationary phase (5 × 10^7^ cells/ml) parasites were extracted in solvent E (H_2_O/ethanol/diethyl ether/pyridine/NH_4_OH; 15:15:5:1:0.017); dried under a stream of N_2_; resuspended in 0.1 n acetic acid, 0.1 m NaCl; and applied to a column of phenyl-Sepharose (1 ml), equilibrated in the same buffer. LPG was eluted with solvent E. The PG repeats of LPG were generated by depolymerization under mild acid conditions (0.02 n HCl, 15 min at 60 °C) and dephosphorylated with *E. coli* alkaline phosphatase (5 units/ml, 16 h at 37 °C) (7, 65). Aliquots of dephosphorylated PG repeats were fluorophore-labeled at the reducing ends with 8-aminonaphthalene-1,3,6-trisulfate and analyzed by FACE according to the manufacturer's specifications (Glyko Inc., Novato, CA).

Purified LPG was resolved by 12% SDS-PAGE and electroblotted onto Hybond ECL nitrocellulose membranes (Amersham Biosciences). Mouse monoclonal antibody WIC79.3 was used to detect LPG (1:1000 dilution) (66). An enhanced chemiluminescence detection system (Amersham Biosciences) was used to detect signal.

### Double knock-out A/FKPs in the presence of ectopically expressed AFKP80

The ORF was released from pXG*-AFKP80* (B5992) by digestion with SmaI and blunt-ligated into the BglII site of pXNG5-Phleo (B6432), yielding pXNG5-Phleo-AFKP80 (B6481). The construct was transfected into Δ*afkp80*^−^ mutants, yielding Δ*afkp80*^−^/+pXNG-*AFKP80*, whose activity was confirmed by 3F12 agglutination. The cell was further submitted to third- and fourth-round replacements with *FKP40*::*BSD* and *FKP40*::*SAT*, resulting in Δ*fkp40*^−^/Δ*afkp80*^−^/+pXNG-*AFKP80*.

### Single-cell sorting

Before cell flow cytometry, Δ*fkp40*^−^/Δ*afkp80*^−^/+pXNG-*AFKP80* cells were grown in M199 without any drug for 24 h, washed with PBS, and filtered through CellTrics 50-μm filters (Partec). Single-cell sorting was then performed based upon their GFP fluorescence using a Dako MoFlo high-speed cell sorter, with single cells selected by stringent gating on forward- and side-scatter parameters. Single cells were placed into individual wells of 96-well plates, each containing 150 μl of M199 medium. Plates were incubated at 26 °C for 2 weeks, and parasite growth was scored.

### Cloning and overexpression of TbGMD and TbGMER in L. major

GMD and GMER ORFs (GenBank^TM^ accession numbers AM746334 and AM746335) were amplified by PCR from genomic DNA of *T. brucei* 427 using primer pairs with XmaI or BglII restriction sites added (SMB 3448/3449 for *GMD*, SMB 3450/3451 for *GMER*). PCR products were digested with XmaI (*GMD*) and BglII (*GMER*), ligated into the XmaI and BglII sites of pIR1Phleo (B6175), respectively, producing pIR1Phleo-*TbGMD-GMER* (B6339). The construct was electroporated into the third targeting round (“3/4”) *afkp80*^−^/Δ*fkp40*::*BSD*/*FKP40*, resulting in *afkp80*^−^/Δ*fkp40*::*BSD*/*FKP40*/+*TbGMD-GMER.* The resulting cell was then submitted to the fourth-round replacement with *FKP40*::*SAT* to replace the last copy of *FKP40*, yielding *fkp40*^−^/*afkp80*^−^/+*TbGMD-GMER*.

### Nucleotide-sugar analysis

Sugar nucleotide extraction and analysis were performed as described (27). Briefly, cells were pelleted by centrifugation, washed in ice-cold PBS, and lysed in 70% ethanol in the presence of 20 pmol of the GDP-glucose as an internal standard (Sigma). The lysate was centrifuged to remove insoluble material, and the supernatant was extracted with butan-1-ol to remove lipids. Sugar nucleotides were extracted from the resulting aqueous phase using EnviCarb graphitized carbon columns (Supelco) as described previously (67). The eluted sugar nucleotides were analyzed by multiple-reaction monitoring LC-MS/MS (27).

### Electron microscopy

Parasites were fixed in 4% paraformaldehyde, 0.05% glutaraldehyde (Polysciences Inc., Warrington, PA) in 100 mm PIPES, 0.5 mm MgCl_2_, pH 7.2, for 1 h at 4 °C. Samples were then embedded in 10% gelatin and infiltrated overnight with 2.3 m sucrose, 20% polyvinylpyrrolidone in PIPES/MgCl_2_ at 4 °C. Samples were trimmed, frozen in liquid nitrogen, and sectioned with a Leica Ultracut UCT cryo-ultramicrotome (Leica Microsystems Inc., Bannockburn, IL). 50-nm sections were blocked with 5% FBS, 5% normal goat serum and subsequently incubated with biotinylated UEA-I (1:20) (Vector Laboratories, Inc., Burlingame, CA) followed by streptavidin conjugated to 15-nm colloidal gold (BB International, Cardiff, UK). Sections were washed in PIPES buffer, followed by a water rinse, and stained with 0.3% uranyl acetate, 2% methyl cellulose. Samples were viewed with a JEOL 1200EX transmission electron microscope (JEOL USA Inc., Peabody, MA). Parallel controls omitting the biotinylated UEA-I were consistently negative at the concentrations of streptavidin used.

## Author contributions

H. G., M. A. J. F., S. J. T., and S. M. B. designed the study; H. G., N. M. N., and G. B. performed the experiments; all authors were involved in data analysis; and H. G. and S. M. B. wrote the paper.

## Supplementary Material

Supplemental Data

## Footnotes

4 N. M. Novozhilova, T. Notton, H. Guo, S. J. Turco, and S. M. Beverley, manuscript in preparation.

## Footnotes

3 The abbreviations used are:

LPG: lipophosphoglycan
PG: phosphoglycan
Fuc: l-fucose
Man: mannose
FKP: fucokinase-pyrophosphorylase
A/FKP: arabino/fucokinase-pyrophosphorylase
FACE: fluorophore-assisted carbohydrate electrophoresis
nt: nucleotide(s)
d-Ara*p*: d-arabino*pyranose*
UEA-I: *U. europaeus* agglutinin I
FU: fluorescence units
GMD: GDP-mannose 4,6-dehydratase
GMER: GDP-fucose synthetase
LmFV1: *L. major* strain Friedlin V1.
