# Supplementary material for: Genetic metabolic complementation establishes a requirement for GDP-fucose in Leishmania
Source: J Biol Chem. 2017 May 2;292(25):10696–708. doi: 10.1074/jbc.M117.778480 (PMC5481574; doi:10.1074/jbc.M117.778480)
Supplement: Supplemental Data [file supp_292_25_10696__index.html]

Genetic Metabolic Complementation establishes a requirement for GDP-Fucose in Leishmania — Genetic metabolic complementation establishes a requirement for GDP-fucose in Leishmania — GDP-fucose is an essential metabolite for Leishmania — Supplemental Data 

# Genetic metabolic complementation establishes a requirement for GDP-fucose in *Leishmania*

## Supplemental Data

- Supplemental info (.pdf, 207 KB) - Supplemental info - text
- Supplemental Figs (.pdf, 166 KB) - Supplemental figures
