# Supplementary material for: Genetic metabolic complementation establishes a requirement for GDP-fucose in Leishmania
Source: J Biol Chem. 2017 May 2;292(25):10696–708. doi: 10.1074/jbc.M117.778480 (PMC5481574; doi:10.1074/jbc.M117.778480)
Supplement: Supplemental Data [file 10.1074_M117.778480_jbc.M117.778480-1.pdf]

April 20 2017

Supplemental information for **"Genetic Metabolic Complementation Establishes a Requirement for GDP-Fucose in *Leishmania*",** by Hongjie Guo, Natalia M. Novozhilova, Giulia Bandini, Salvatore J. Turco, Michael A. J. Ferguson, and Stephen M. Beverley

SUPPLEMENTAL FIGURES

Figure S1. PCR confirmation drug marker replacements within *A/FKP80* single gene knockouts. Panel A shows the map of the planned *AFKP80::HYG* and *AFKP80::PAC* replacements, and **Panel C** shows the map of the planned *FKP40::BSD* and *FKP40::SAT* replacements. The large grey boxes depict the *HYG*, *PAC*, *BSD* and *SAT* ORFs, the narrow flanking grey and white bars depict regions conserved between *A/FKP*s on the 5' and 3' sides respectively, and the narrow black bar depicts the extent of the targeting fragments. The analysis of the *A/FKP* coding regions is shown in Figs. 2B, C. **Panel B**. PCR confirmation of planned integration of drug resistant markers in a representative Δ*afkp80*$^{-}$ mutant. Primers outside the 5'- and 3'-flanking regions used for gene replacement and primers inside drug marker ORFs were used for PCR to confirm the integration of the drug marker ORFs into the chromosome. Primers a/e and d/f establish the left (5') and right side (3') of *PAC* replacement (*ℓ PAC*/*r PAC*), and primers a/b and d/c establish the left (5') and right side (3') of *HYG* replacement (*ℓ HYG*/*r HYG*). Primer a, SMB2796; e, SMB 2889; f, SMB2888; b, SMB2566; c, SMB2565; d, SMB2793. **Panel D**. PCR confirmation of planned integration of drug resistant markers in a representative Δ*fkp40*$^{-}$ mutant. Primers g/l and k/j establish the left (5') and right side (3') of *SAT* replacement (*ℓ SAT/r SAT*), and primers g/h and i/j establish the left (5') and right side (3') of *BSD* replacement (*ℓ BSD/r BSD*). Primer g, SMB2783; l, SMB 2768; k, SMB2769; h, SMB2555; i, SMB2556; j, SMB2784.

Figure S2. The size of LPG in logarithmic and stationary phase is unchanged in the arabinose-deficient Δ*afkp80*$^{-}$ mutant. A western blot with antibody WIC79.3 of LPG purified

from logarithmic or stationary growth phases from both WT and Δ*afkp80*⁻ is shown. WIC79.3 recognizes Gal-modified LPG (66).

Figure S3. Attempts to generate *A/FKPs* double mutants by successive rounds of replacement yielded 4 successful replacements but retention of *FKP40*. Panel A. Planned disruption of *FKP40* by homologous gene replacement by methods depicted in Fig. 2, S1. In a third round of gene replacement, the Δ*afkp80*⁻ mutant was transfected with *FKP40::BSD*. PCR tests (not shown) confirmed this was successful, yielding a line lacking 3 of the 4 *A/FKP* alleles (Δ*afkp80*⁻/*FKP40::BSD*/*FKP40*). In a fourth round, this line was transfected with the *FKP40::SAT* targeting fragment, with the expectation of yielding homozygous *A/FKP* double mutants. **Panel B**. PCR tests established that this line lacked *AFKP80* (lanes labeled *r AFKP*), but retained *FKP40* (*r FKP* lanes) despite the presence of the planned *FKP40::BSD* and *FKP40::SA*T replacements (lanes labeled ***ℓ*** *BSD*, *r BSD*, ***ℓ*** *SAT* and *r SAT*). See Fig. S1 for location of primers used in PCR tests and not shown are data for the *AFKP80* replacements (Figs. 2, S1).

Supplemental Table.

Table S1. Oligonucleotides used in this work.

Primer	Sequence
SMB2446	AGCTGCGCGACGCTGAGTTCCTGCACTA
SMB2450	GATTGAGCAGATGCTGACTGCTGGT
SMB2451	TACATGTACATGTGCGCGAAGGACGAG
SMB2453	GACAGGAACGACCTCTTC
SMB2555	**GGTAACGGTGCGGGCTGACG**CCACCATGGGCCAAGCCTTTGTCTCA
SMB2556	**CGAGATCCCACGTAAGGTGC**TTAGCCCTCCCACACATAACCAGAG
SMB2557	**GGTAACGGTGCGGGCTGACG**CCACCATGACCGAGTACAAGCCC
SMB2558	**CGAGATCCCACGTAAGGTGC**TCAGGCACCGGGCTTGCG
SMB2561	**GGTAACGGTGCGGGCTGACG**CCACCATGAAAAGCCTGAACTC
SMB2562	**CGAGATCCCACGTAAGGTGC**CTATTCCTTTGCCCTCG
SMB2565	AATACGAGGTCGCCAACATC
SMB2566	GAAAGCACGAGATTCTTCGC
SMB2630	**GGTAACGGTGCGGGCTGACG**CCACCATGAAGATTTCGGTGATCC
SMB2631	**CGAGATCCCACGTAAGGTGC**TTAGGCGTCATCCTGTGCTCC
SMB2664	CATGCCATGGGGCGAAACAGCGACCACA
SMB2665	**CGTCAGCCCGCACCGTTACC**CGTGGCACACGGCTCTGTG
SMB2666	**GCACCTTACGTGGGATCTCG**AGAGGCGTGCGGCGGTGTG
SMB2667	GGGAATTCCATATGCGCCTCAAGCTCTACCCACC
SMB2668	CATGCCATGGCATCCCTGCCGAACGCCGAACC
SMB2669	**CGTCAGCCCGCACCGTTACC**GGTTGCAGGGGAGGCTGAAGA
SMB2670	**GCACCTTACGTGGGATCTCG**TCTTAGAGGCGTGCGGCGGT
SMB2671	GGGAATTCCATATGGAGGTGCGATGCGGTGTTCGGGGTT
SMB2768	CTTGGTCGGATAGGTGCAC
SMB2769	AGCGATGTACTGGTACTGG
SMB2783	CATAGCAGCTGAGCAGCAG
SMB2784	TGGTGCGACGCAGCCGTAG
SMB2793	AGATGAGGAACCTAGAGAG
SMB2796	CCCGTATTCCTTGTCCTTTCATGGTACC
SMB2828	TCCCCCCGGGCCACCATGAACGTGAGCTTTCTCTTCAGCC
SMB2829	TCCCCCCGGGCTAAGACCGGCTGATCTGGAGGCCGGACG
SMB2830	GGAGCTGATGCGATGCTCTCC
SMB2889	ACCGTGGGCTTGTACTCGG
SMB2890	ATTGTTCAGGCTTCAAGCGACC
SMB3176	CAGCAGCCCTCTATATACCCGC
SMB3448	TCCCCCCGGGCCACCATGGAACAAAAACTCATCTCAGAAGAGGATCT<br>GTCAGCACGTCGACTGGC
SMB3449	TCCCCCCGGGTATTGCCCCGCTGCTCAACAC
SMB3450	GGAAGATCTCCACCATGTACCCATACGATGTTCCAGATTACGCTTTAG<br>GGTCCCTACCGAGT
SMB3451	GGAAGATCTTCACTTCCGTGCGACATCGTAGTT

SMB3522	CCAAGAGGTGTCTAATCGCTGC
SMB3523	GACTGGACAGTCAACGATCCGC
SMB3524	GCGGATCGTTGACTGTCCAGTC
SMB3525	GAAGAAGCAGCTCATCAAGTGG
SMB3526	CCACTTGATGAGCTGCTTCTTC
SMB3527	ACGCACCCTCTCTAGGTTCC
SMB3752	AACTGCTGCTGCAGCAGCGCAACC

Restriction sites are underlined and linker sequences are in bold.
