# Supplementary figures and images for: Genetic metabolic complementation establishes a requirement for GDP-fucose in Leishmania
Source: J Biol Chem. 2017 May 2;292(25):10696–708. doi: 10.1074/jbc.M117.778480 (PMC5481574; doi:10.1074/jbc.M117.778480)

Figure S1

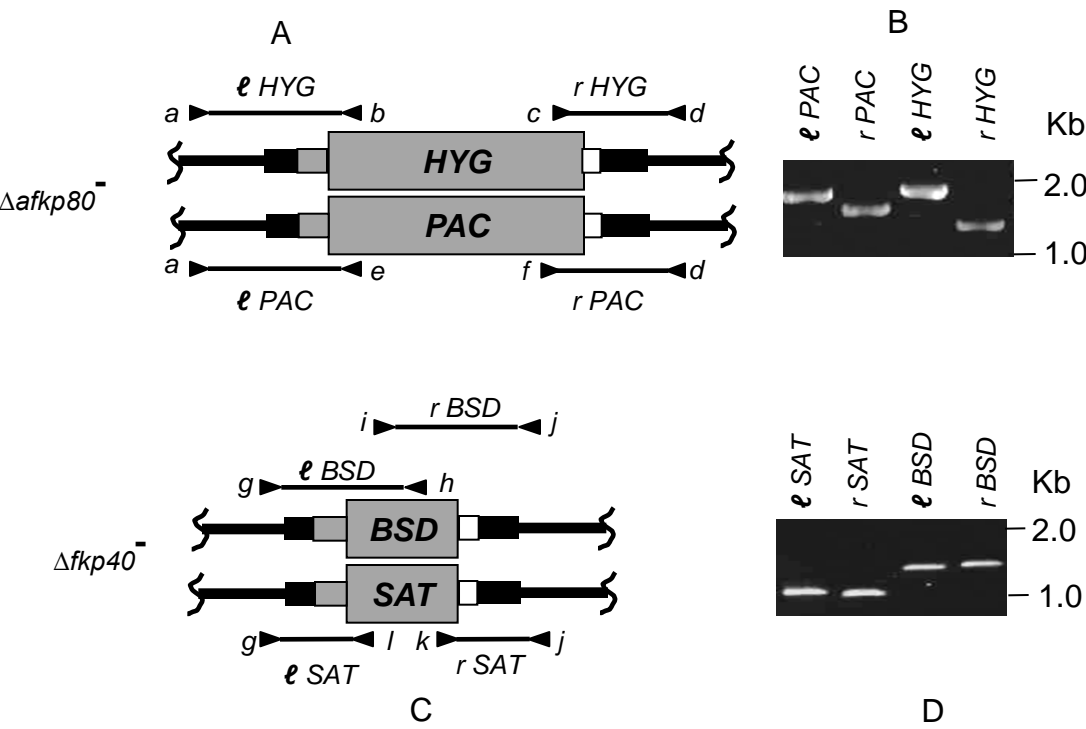

Figure S2.

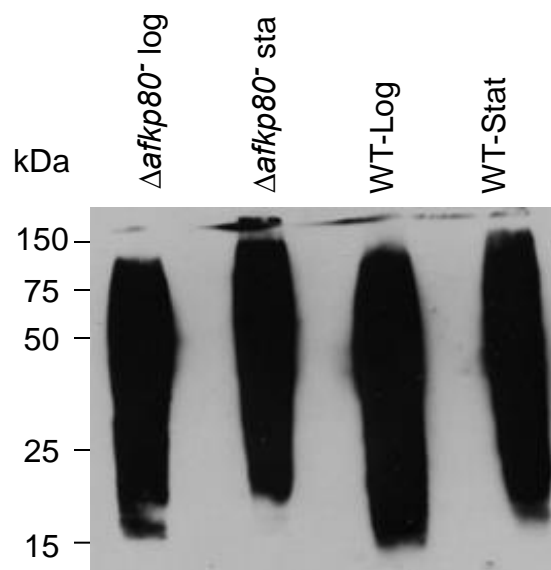

**Figure S3.**

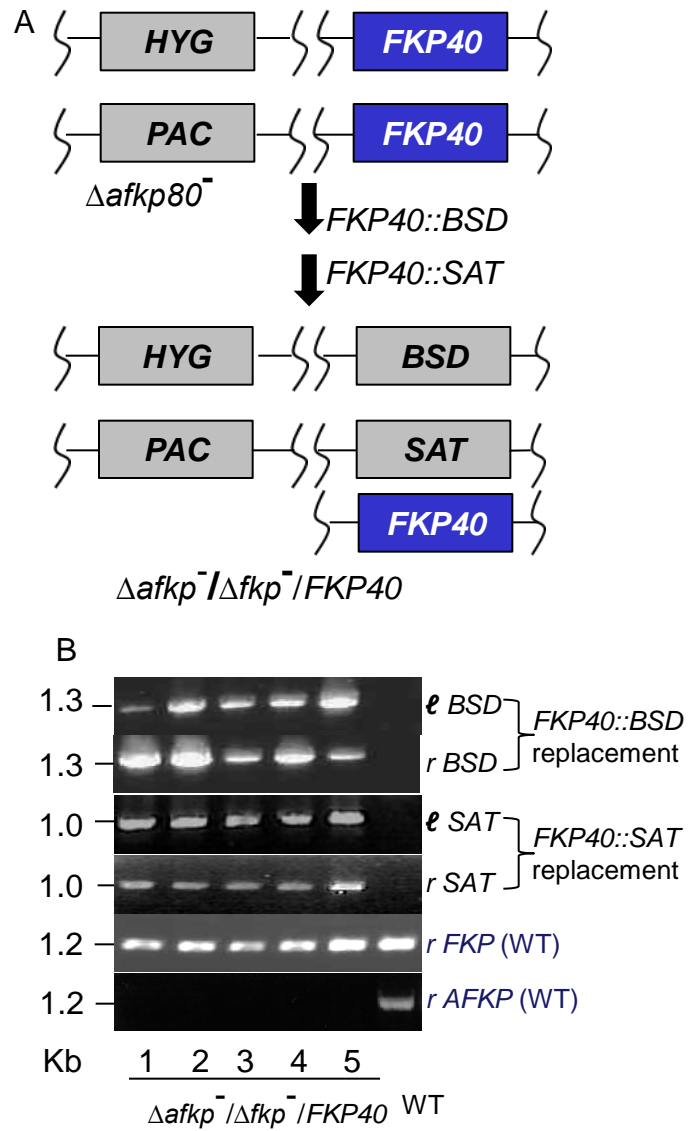

Supplement: Supplemental Data [file 10.1074_M117.778480_jbc.M117.778480-2.pdf]
